# The Trichina Spiralis

**Published:** 1866-05

**Authors:** D. W. Flora

**Affiliations:** Chicago, Illinois


### SELECTIONS.

*The Trichina Spiralis.*—By Dr. D. W. Flora, Chicago, Illinois.

Man in all ages has dreaded the "worm" which destroys the body after death, but the "worms" which prey upon the body while living, should give him most concern. These parasites not only find their way into his body soon after birth, but are found in the fœtus in utero!

Many species crawl upon the epidermis, hide in its hairy covering or pierce through it and burrow and propagate in the cutis vera or the cellular tissue. These, on account of their habit, are called *Epizoa*. A still larger number inhabit the interior of the body. Not one of the internal viscera is exempt. No less than five species of vermes inhabit the alimentary canal.

The heart, brain, liver, lungs, spleen, kidneys, bladder, and eye has each its peculiar species of parasite. These form the other grand division called *Entozoa*.

Belonging to the latter division is found still another species of parasite which has proven a more deadly foe to man than any or perhaps all the others. This is the terrible *Trichina Spiralis*, and its history and the ravages which it has caused in the animal body form the subject of this article.

Authors on Natural History place it in the class, *Nematodes*. It is a microscopic parasite, and presents a striking contrast with the Guinea worm and Tape worm which exceed a score of feet in length.

It was first discovered in the year 1832, but was not scientifically described until 1835, when Prof. Richard Owen, the eminent zoologist, gave to the world its natural history. It was named the trichina spiralis from its resemblance to a fine hair coiled upon itself, like a watch-spring. During that year six persons were admitted to the London hospitals, who were the subjects of trichina poisoning. Since that time the leading medical men have been on the look-out for this parasite, and several cases have been observed and fully described. This parasite is only visible to the naked eye when enclosed in its chalky capsule. These appear as small white points, when a section of muscle is examined.

Since the investigations of Owen, many eminent German physicians and men of science have given attention to the subject, among whom we find Virchow, Herbst, Kuchenmeister, Zenker, Bischoff, and, more recently, Prof. Leukhardt, of the University of Giesen, who undertook a series of scientific experiments with this parasite, and to his extended and systematic observations, we are indebted for the very accurate knowledge we possess of this animal. By introducing it into the stomach of the dog, cat, rabbit, fowl, dove and crow, he was enabled to observe all the phenomena of the trichina disease. Out of nine dogs thus treated, *seven* died, and the muscles were afterwards found *alive*, with free trichina. Of the other animals subjected to similar experiments, more than one-half died. The impression pretty generally prevails that the *fowl* is not subject to this disease, the trituration or grinding in the gizzard being sufficient to destroy the animal. The reverse of this is most probably true, as the grinding process only aids the solvent power of the gastric fluid in dissolving the calcareous shell.

The trichina is usually introduced into the stomach of a man in its capsule, though it is possible for it to find its way there in the free state. This calcareous shell is dissolved by the acid juices of the stomach, and the parasites thus set free rapidly acquire their growth and sexual power, and copulation and reproduction are perfected within one week after their introduction into the alimentary canal. The female brings forth from 60 to 100 live trichinæ.

The young swarm immediately commence their work of destruction by piercing the coats of the stomach and the intestines. It is not definitely known how long the parent trichinæ live after producing the new swarm; but it is certain that they never leave the stomach and alimentary canal, and probably die within a fortnight. The instincts of the young trichinæ impels them to seek the fibre of the *voluntary* muscles, which is known to the anatomist as the striated muscular fibre.

The *brain*, *lungs* and *liver* they avoid, and but a single trichina has been found in the substance of the *heart*. The wonderful instinct of this parasite is equal to that shown by the larva of the ichneumon fly, which is hatched in the body of the caterpillar and other grubs, and there devours the cellular and muscular tissue, but carefully avoids the nervous and circulatory systems which are essential to life. In this way the fable of Promethus is realized, the poor "worm" eating and growing, only to be continually consumed.

In order to reach the muscles, the coats of the stomach and intestines must first be pierced, and this boring operation gives rise to a train of symptoms which closely resemble acute diarrhœa and dysentery, as bloody stools frequently occur. Ulceration and perforation of the bowels are frequent results of this inflammation. When the serous membrane lining the abdominal walls is attacked, the symptoms of peritonitis is added. The inflammation of the serous membrane, if it does not prove fatal, leaves the intestines firmly glued together. If the agonized patient should live through these primary acute forms of disease, a new train of symptoms, closely resembling typhus or septic fever, will be ushered in. To diarrhœa, dysentery and peritonitis, will be added intense pain in the muscles, great nervous irritability from prostration, spasm of the muscles, and finally paralysis and death. The muscles of the abdomen, of course, are first attacked, but the trichinæ soon find their way to those of the chest and anterior portion of the neck, and by degrees reach the muscles of the back and extremities.

The track of the trichina may be seen as indicated in the last engraving by the white line or hand, which was once a striped muscular fibre, but which is now only "detritus," or digested muscular tissue, in which the full grown larva may be seen elaborating its shell, preparatory to entering the pupa or quiescent state. In this stony house the self-immured trichina remains, without doubt, so long as the animal into whose body it has found its way continues to live, which may be decades of years. In this state they are sexless, and the further propagation of the species seems to depend solely upon the accidental circumstance of this parasite finding its way into the stomach of some living animal, where it may develop its sexual characteristics, and multiply its species.

In the transformations of this parasitic animal may be seen many analogies to insect life. The period of wandering and feeding is the "larva" stage. The encapsuled trichina is the "pupa" or "chrysalis," in which state it remains until released in the manner already described. The muscles upon which this animal has fed become useless in proportion to the number of ultimate fibres which are destroyed. When it is remembered that a single ounce of flesh may contain trichina enough to produce in eight days 3,000,000 young, it is not surprising that the entire substance of the abdominal muscles should be sometimes found consumed.

The symptoms progress, and the terrible fatality of this disease is well exemplified in the history of the Hettstadt tragedy, which is taken from a British medical journal:

"The village is situated near the Hartz mountains, in Germany. An annual festival was celebrated there some two years since, and one hundred and three persons sat down to a dinner, the third course consisting of *roste-wurste* and *gemuse*, (sausage and vegetables.) The sausage had been prepared beforehand for this special occasion. The steward, who had been commissioned to furnish the pig for this purpose, gave the butcher a lean, ill-conditioned one, instead of the thrifty one which had been bargained for. The day after the festival, several persons who had participated in the dinner were attacked with pain and irritation of the intestines, with loss of appetite, fever and great prostration. The number increased from day to day, and an epidemic of typhus or septic fever was apprehended, as the symptoms began to assume that character. However, as the disease progressed, the symptoms assumed a different type, and to diarrhœa, dysentery and fever, were added peritonitis, circumscribed pneumonia and paralysis of the abdominal and intercostal muscles with those of the neck. Then the typhus theory was abandoned, and some unknown poison was assumed to be at the bottom of it. Under this conviction, every article of food and material used in connection with the dinner was rigidly examined. By this time the trichinæ had reached the muscles of the calf of the leg in some of the victims, and Zenker's description of the disease was called to mind. The remnants of the sausage were examined, and found to be literally 'swarming' with trichinæ. Portions of muscle from the calf of the leg of the affected ones were examined under the microscope, and were found full of *free* trichina. These were the progeny of the *encapsuled* ones which had escaped the smoking and frying process to which the sausage had been subjected.

"No less than eighty-three of the above-mentioned number died within a few weeks, and the surviving twenty at last accounts were still lingering in agony, and apprehensive of a similar fate.

"This awful catastrophe at Hettstadt awakened sympathy and fear throughout all Germany, and many eminent medical men were consulted in the interest of the sufferers, but none could bring relief or cure. With an obstinacy unsurpassed by any other disease, trichiniasis surely carried its victims to the grave.

"Many *vermifuges* were employed with the hope of removing the parasites still in the alimentary canal. Picric acid was employed, until its effects seemed as dangerous as the disease itself. An examination of the bodies after death showed the trichinæ to have been unaffected by any of the remedies employed. The terrible conviction now fastened itself upon the minds of all who witnessed these scenes, that a person afflicted with this parasite was doomed to die the slow death of exhaustion from nervous irritation, fever and paralysis of all the muscles."

Dr. W. Mueller, of Homberg, was summoned to the place (Hettstadt) to attend a relative who had been poisoned by this trichinous sausage. "On my arrival," he says, (Nov. 11th,) "I found the patient—who, previous to the attack, was a strong and healthy man, twenty-three years of age—perfectly conscious, with a slight œdematous swelling of the face.

On examination of the chest, a dull sound over about one inch and a half of the lowest part of the lower lobe or left lung was produced by percussion; crepitating rattles were audible, but there was no bronchial breathing, thus showing the beginning of resolution of the pneumonia; at the lowest part emitting the dull sound, there was a slight pleuritic rubbing. The pulse was 140; respirations 48; and the temperature of the body 39° centigrade. The symptoms of the disease commenced on the 16th of October, with loss of appetite and diarrhœa, followed by a sensation of painful weakness in the limbs and difficulty in moving the tongue, the pulse being above 100.

The patient was not confined to his bed during the day-time until the 6th of November, when the pneumonic symptoms commenced. The day after my arrival, (November 11th,) the symptoms were unaltered, with the exception of the pleuritic rubbing, which had moved a little higher up.

The whole of the pleuro-pneumonic affection was so very trifling that it certainly did not account for a pulse of from 140 to 150, and for the violent oppression, or rather as the patient explained it himself, "the weakness in drawing his breath."

The day following, the frequency of respiration varied between 30 and 60; the pulse was more than 200 and very weak; the temperature had fallen to 38° 6′ cent.; and the body was covered with a profuse clammy perspiration.

The other physical symptoms were as before, and the pleuritis had not extended higher. The complaint of weakness in breathing, or as the patient called it, "the impossibility of drawing a sufficient quantity of air into the lungs," was increased; but he remained conscious and resigned, so much so that he several times asked me at what hour I expected he would die. At 7 o'clock P. M., November 12th, he died. The post mortem examination, performed on the 13th, proved an infiltration of a part of the lower lobe of the left lung, extending upwards about an inch and a half from its lower margin, and three or four ounces of liquid exudation were found in the pleural cavity of the same side. When examining the chest and intercostal muscles, I found in every small piece placed under the microscope, trichinæ partly wound up, but not capsulated, partly forming a single sling and partly extended. In the examined parts of the heart and diaphragm, no trichinæ were discovered.

The piric acid before mentioned, which was so heroically applied in the treatment of the human subject, was administered to a pig infested with trichinæ. The animal died from its effects, but the trichinæ were found alive and unhurt on examining its body.

Prof. Mosler had an opportunity during the epidemic of trichiniasis which occurred at Quedlinburg, in another part of Germany, of trying the efficacy of *benzine* in this disease. It was first tried on the lower animals, and the success which attended its administration, induced him to try it upon man. The form in which it was given was, Benzine, ʒ ij.; Sol. Liquorice, Muc. Ac. aa., ℥ j.; Aqua Menth. ℥ iv. Of this mixture a tablespoonful was given every hour or two.

The conclusions arrived at by Dr. Mosler in regard to this remedy, are as follows: "Benzine holds the first place among anthelmintic remedies, and may be administered in larger doses to the human subject than was formerly thought possible; given in doses which are well borne by the human subject, it kills the trichinæ in the intestinal canal and thus prevents its propagation or the possibility of reaching the muscular tissue.

Since the Hettstadt tragedy, the public mind in Germany has had little rest from apprehension of this terrible scourge. A wholesale poisoning soon after occurred in Offenbach, a manufacturing town in Hesse Darmstadt. Upwards of twenty persons were poisoned by eating trichinous pork, several of whom have died. But Hettstadt, with its tragedy and appalling concomitants, is eclipsed by the late visitation at Hedersleben, another German village, where three hundred inhabitants partook of trichinous pork, and at this writing full one hundred are in their graves. The butcher slaughtered four pigs, which were sold to the villagers. The butcher and his wife, partaking of the same meat, became themselves the earliest victims. A very injudicious custom seems to have obtained in this village, as well as in many other parts of Germy, namely: that of eating pork in a raw state, cut fine and spread upon bread. Although the scenes at Hettstadt were still fresh in the public mind, and the very uniform character which the symptoms always present was well understood by the medical profession generally, yet the people, through fear and ignorance, fled from what they believed a visitation of *cholera*.. The consequences can easily be imagined. Many were seized with the disease, and died on the highways. The irritation of the stomach, vomiting and diarrhœa, might well be taken for the premonitory symptoms of the above disease by the mass of the people. Indeed, the village doctor was himself misled, and he treated the sufferers with opium and astringents. This treatment was evidently intended to control the diarrhœa; but it proved fatal to the patients, by confining the parasites in the stomach and alimentary canal until they had an opportunity to pierce through the walls. Out of twenty-eight persons treated in this manner, twenty-seven died. One very remarkable fact has been noted in connection with the epidemic at Hedersleben, namely: that as yet no *children* have died of the disease, all having made a good recovery.

The recent developments in Germany will lead many to inquire anxiously into the history of the trichina in our own conntry. It has been known for some years among medical men that the encapsuled trichina was occasionally found in the muscles of the human body after death, but until very recently no authenticated cases have been reported where death was produced by the disease in question.

In the *American Medical Times* of February 20, 1864, a case is reported by Dr. Schnetter, in which a whole family was poisoned by eating trichinous pork. The father was the only one in which the poison proved fatal. This case occurred in New York city. The *Buffalo Medical Journal* contains the account of two fatal cases occurring in the western part of the State. A man and his wife residing in the village of Chiktonaga were found to be affected by an "apparently acute rheumatism, of a peculiar character." Dr. Krombein, the attending physician, suspected trichinæ, and, the patients having shortly died, a microscopic examination was instituted by Drs. Krombein and Homberger, which demonstrated the existence of the parasite in great numbers. The specimens of muscle taken from the bodies of the dead, together with a remnant of the sausages of which they had partaken, were subsequently examined by Dr. Lathrop and Prof. George Hadley, under the microscope, and trichinæ found in both. In the human muscle they were *free;* in the sausage, *encysted.* Other members of the family were affected, but probably did not eat enough to prove fatal.

All the evidence thus far adduced goes to show that the more the cooking the milder the disease. Those cases in which the pork was eaten *raw*, were of the most violent character, and invariably proved fatal. When it is remembered that this parasite in its capsule has been subjected to a degree of heat little below the boiling point, without destroying its vitality, the importance of *thorough cooking* becomes apparent to the lover of swine flesh.

In examining pork for trichina, the capsules may sometimes be seen by the naked eye under the tongue; but by far the safer plan is to have a magnifying glass of moderate power—say from ten to twenty-five diameters—which reveals whether it be free or encysted.

It is well known that the common red "earth-worm" or "angle-worm," is infested with thrichinæ, and in this way fowls and swine may become the subjects of the disease, as they devour the worm greedily. An opinion obtains with many persons that what is known as "measly pork," is more liable to be infected with trichinæ than any other. What facts there are to sustain this belief, we are not acquainted with, but the "measles" in the hog is genuine *scrofula*, and it is a significant fact that the disease just mentioned should have derived its name from *scrofa*, a *sow*. The ancients, however wild or erroneous may have been their *theories*, were nevertheless close and accurate observers of *facts*. In this way the *name* of the disease is made to indicate its *origin*. It is safe to conclude that more disease and death are caused by eating pork *without* trichinæ than *with* them. To those who are determined to eat swine flesh in spite of the trichinæ and the law of Moses, we would give some advice in regard to the manner of rearing them. "As filthy as a hog," is a common comparison; yet the pig is sometimes libeled. He has his likes and dislikes, and though he seek his food among ordure or in the filthy gutter, yet he will not eat unsound or unhealthy food. If he is shut up in a close pen, and made to swim in his own excrements, he certainly is not responsible for his dirty plight. The fact is now pretty well understood in Germany that the pigs which have been infested with trichinæ were brought up in this manner, and gave evidence of bad health before they were slaughtered.

While engaged in penning this article reports are coming to us daily from different parts of the country of the discovery of trichinous pork, and at Detroit the death of a German lady is reported in whose body this parasite was found. *Thorough examination and thorough cooking* have been insisted upon in this article as preventives of trichina disease; but the only *infallible* one is to obey the Jewish law, and *eat no pork.*—*Cincinnati Lancet & Observer.*

